# Band Gaps and Vibration Isolation of a Three-dimensional Metamaterial with a Star Structure

**DOI:** 10.3390/ma13173812

**Published:** 2020-08-28

**Authors:** Heng Jiang, Mangong Zhang, Yu Liu, Dongliang Pei, Meng Chen, Yuren Wang

**Affiliations:** 1 Key Laboratory of Microgravity, Institute of Mechanics, Chinese Academy of Sciences, Beijing 100190, China; hengjiang@imech.ac.cn (H.J.); liuyu@imech.ac.cn (Y.L.); peidongliang@imech.ac.cn (D.P.); yurenwang@imech.ac.cn (Y.W.); 2 School of Engineering Sciences, University of Chinese Academy of Sciences, Beijing 100049, China; 3 Wuhan Second Ship Design and Research Institute, Wuhan 430064, China; zmg09@tsinghua.org.cn

**Keywords:** metamaterials, Poisson ratio, band gaps, vibration isolation, 3D star structure

## Abstract

Elastic metamaterials have promising applications in wave control and vibration isolation, due to their extraordinary characteristics, e.g., negative Poisson ratio, band gaps, effective negative mass density and effective negative modulus. How to develop new functional metamaterials using a special structure has always been a hot topic in this field. In this study, a three-dimensional (3D) star structure is designed to construct metamaterials with both negative static and dynamic properties. The results show that the 3D star structure formed a wide band gap at lower frequency and had a negative Poisson’s ratio. Different from conventional acoustic metamaterials, the main physical mechanism behind the low-frequency band gap of the 3D star structure is the resonance mode formed by the bending deformation of each rib plate, which made it easier to achieve effective isolation of low-frequency elastic waves with a low mass density. In addition, many structural parameters of the 3D star structure can be modulated to effectively adjust the band gap frequency by changing the angle between the concave nodes and aspect ratio. This study provides a new way to design the 3D acoustic metamaterials and develop the lightweight vibration isolation devices.

## 1. Introduction

Metamaterials are artificial materials consisting of atom-like units arranged periodically and have potential applications in many fields because of their extraordinary characteristics not displayed by natural materials; e.g., negative Poisson ratio [1,2,3], negative effective mass density [4,5,6], and negative effective modulus [7,8,9]. Mechanical metamaterials with a negative Poisson ratio have excellent mechanical and physical properties such as high designability, co-bending, and energy absorption efficiency [10,11,12,13,14], which make them attractive as biological scaffolds [15,16,17,18]. Elastic metamaterials with negative effective density have been designed as vibration isolation structures because of their suitable band gaps [19,20,21]. A current focus of research on metamaterials is the design of new structural materials with multiple negative effective properties to realize comprehensive functions. It is assumed that the combination of mechanical metamaterial and elastic metamaterial designs will lead to metamaterials with not only excellent static mechanical properties, but also directional control over the transmission of elastic and acoustic waves, which will greatly expand the application scope of metamaterials. In addition, how to achieve vibration isolation in low frequency with broadband is a big challenge for elastic metamaterials, and it also needed to introduce new lightweight structures.

Artificial “metaatoms” (microstructure units) are the key to the design of metamaterials with negative effective properties. Unlike traditional composites, the extraordinary properties of metamaterials are usually derived from their microstructure units with special configurations rather than the simple superposition of component materials. The negative Poisson’s ratio of mechanical metamaterials usually comes from their concave configuration [22,23,24,25] or chiral structure [26,27,28,29,30]. In order to achieve the negative effective density of elastic metamaterials, lumped mass is introduced into a subwavelength unit [4], resulting in dipole resonance. To obtain negative modulus, a rotating resonator [7] is usually introduced into the subwavelength unit, resulting in monopolar resonance. Lattice structures composed of an interconnected network of elastic beams, such as Kagome lattices [31], re-entrant grids [32], and zigzag lattices [33,34], not only possess the negative Poisson’s ratio of mechanical metamaterials, but also have the band gap of elastic metamaterials. Ruzzene et al. studied the band gap characteristics of concave hexagons and noted that a concave structure is conducive to the generation of a directional band gap. Spadoni et al. [30] investigated the band gap characteristics of periodic chiral hexagons. They confirmed that the rotation deformation of chiral hexagons can produce a low-frequency full band gap. Subsequently, Liu and colleagues introduced resonant elements into chiral hexagons to generate a full band gap at low frequencies and analyzed the vibration mode [31]. Meng’s group analyzed the equivalent mechanical behavior and band gap properties of star structures, revealing that the structures contained many broad band gaps at low frequency [35]. In our previous work, a single-phase solid lens with double-negative index was designed using a star structure [36,37]. However, the above metamaterials usually possess two-dimensional (2D) lattice structures and their band gaps are usually directional, which is not practical. Therefore, it is necessary to design a three-dimensional (3D) structure based on the knowledge obtained from previous research to build metamaterials with both static and dynamic negative properties.

In design of the metamaterials with both negative static and dynamic properties, the concave configuration is considered first. To achieve a complete band gap, the unit structure should have a high symmetry such as a simple cubic lattice [38,39]. So, the 3D star structures are chosen to investigate in this paper. Star structural materials possess a typical lattice structure and negative properties arising from the concave configuration of the stars. The mechanical properties of such materials are controlled by the geometrical features of the stars, such as thickness, length, and concave angle. In this paper, a 3D star structure is designed to construct metamaterials with both static and dynamic negative properties. The calculated results suggest that the 3D star structure has a negative Poisson’s ratio when the concave angle is less than 70°. In addition, the star metamaterials possess a lower band gap because of local resonances, which means that they can be used as vibration isolation structures.

## 2. Design of 3D Star Structure and Numerical Calculation Methods

### 2.1. Design of 3D Star Structure

**Figure 1:**
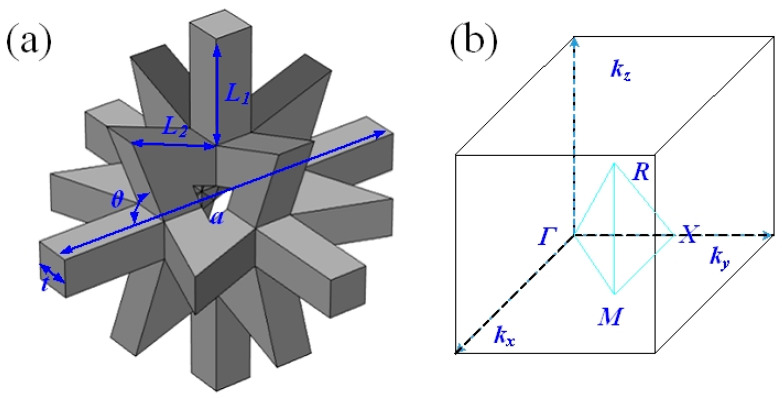
Schematic of the 3D Star structure (**a**) and the Brillouin zone of simple cubic lattice (**b**).

The 3D star structure can be obtained by three orthogonal star sheets, as shown in Figure 1. There are two kinds of beams with the same cross-sectional area and thickness *t* in the unit, they are 6 straight ones with length *L*_1_ and 32 star cannot concave ones with length *L*_2_. The counterclockwise angle between the adjacent cell walls is denoted as *θ*. The 3D star structure is arranged as a cubic lattice with lattice constant a, which can be expressed as a = $2\left\{\left(\sin\left(\theta -45{}^{\circ}\right)\right)/\left(\sin\left(45{}^{\circ}\right)\right)\cdot L_{2}+L_{1}\;\right\}$. Similar to other 3D phononic crystals with ultra-wide band gap [38,39], the 3D star structure also only needs single-phase material. Furthermore, 3D star structure is simpler and lightweight due to the high porosity.

According to the symmetry of the 3D star structure, its properties including the Poisson’s ratio and elastic modulus are the same in the *x*, *y,* and *z* directions. In other words, uniaxial loading of the cell along the *x* direction leads to the same deflection along both *y* and *z* directions. Additionally, uniaxial loading along the *y* or *z* direction results in the same deformation. Therefore, only one direction needs to be considered in the calculation process. This study focuses on the static mechanical properties and wave mechanical behavior of the star structure, which was made of acrylonitrile—butadiene—styrene plastic with a density *ρ* of 1.05 g/cm^3^, Young’s modulus *E* of 2.2 GPa, and Poisson’s ratio *ν* of 0.394. In addition, the properties of materials may affect the frequency of the band gap and vibration isolation.

### 2.2. Calculation of Poisson’s Ratio

**Figure 2:**
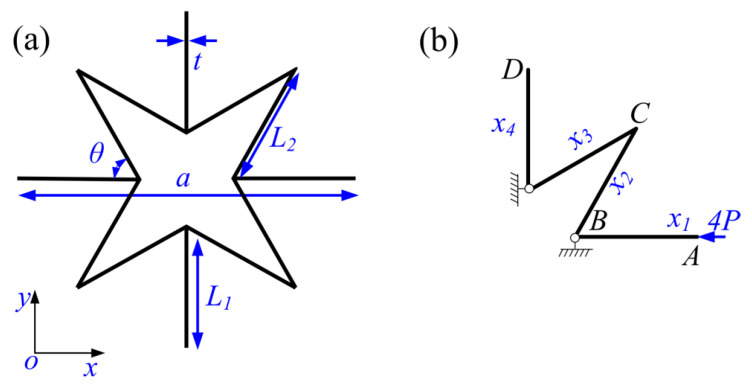
(**a**) The simplified 2D star structure and (**b**) the equivalent representative connected walls under horizontal force *4P* acting at point *A*.

To study the static mechanical properties of the 3D star unit, *ν* was calculated using the force method and finite element method (FEM) [35,40]. Because of the structural symmetry, the force and displacement were the same in the *x*, *y,* and *z* directions, which allowed the structure to be simplified to the 2D structure shown in Figure 2a. Furthermore, the stress and deformations were able to be calculated using one quarter of the unit cell, as shown in Figure 2b, when an axial force was applied to the structure in the *x* direction. The detailed derivation of the internal forces of the static structure in Figure 2b using the force method is presented in Appendix A. Furthermore, the equivalent Poisson’s ratio *v*_12_ is:

(1) $$\nu_{12}=-\frac{\varepsilon_{y}}{\varepsilon_{x}}=-\frac{\frac{PL_{1}{}^{3}}{EI_{1}}(\frac{1}{32}-\frac{5}{48}\sin\theta \cos\theta )+\frac{PkL_{1}}{2GA_{1}}\sin\theta \cos\theta -\frac{2PL_{2}}{EA_{2}}\sin\theta \cos\theta }{\frac{PL_{2}{}^{3}}{EI_{2}}(\frac{5}{96}-\frac{1}{16}\sin\theta \cos\theta )+\frac{kPL_{2}}{4GA_{2}}+\frac{4PL_{1}}{EA_{1}}+\frac{PL_{2}}{EA_{2}}}$$

When there is zero shear in the Euler beam model, *k* = 0, $e=\frac{L_{2}}{L_{1}},f=\frac{t}{L_{1}}$, which means that *v*_12_ can be described by:

(2) $$\nu_{12}=-\frac{\varepsilon_{y}}{\varepsilon_{x}}=\frac{(\frac{3}{8}-\frac{5}{4}\sin\theta \cos\theta )e^{3}-2ef^{2}\sin\theta \cos\theta }{(\frac{5}{8}-\frac{3}{4}\sin\theta \cos\theta )e^{3}+4f^{2}+ef^{2}}$$

When Poisson’s ratio was calculated using the FEM, the 3D star structure was loaded along the *x* direction with the normal deflections. The resulting normal (in *x* direction) and transverse (in *y* and *z* direction) strains could then be obtained by integrating the force and displacement at each boundary.

### 2.3. Calculation of Wave Mechanical Behavior

Wave mechanical behavior of 3D Star structure such as band structure, vibration modes, and transmission loss can be studied using the finite element method (FEMs) with the software COMSOL Multiphysics. The governing equation of elastic wave propagation in solids is given by

(3) $$\rho \frac{\partial^{2}u_{i}}{\partial t^{2}}=\overset{3}{\underset{j=1}{\sum }}\frac{\partial }{\partial x_{j}}\left(\overset{3}{\underset{l=1}{\sum }}\overset{3}{\underset{k=1}{\sum }}c_{\mathrm{ijkl}}\frac{\partial u_{k}}{\partial x_{l}}\right),\left(i=1,2,3\right)$$

where *ρ* is the density of material, *u*_i_ is the displacement, *t* is time, *C_ijkl_* denotes elastic constants of materials, and *x_j_* denotes the coordinate variables *x*, *y,* and *z*. Here, the displacement varies is assumed harmonically over time. Furthermore, the displacement *u*(*r*) can be described as:

(4) $$u\left(r\right)=u_{k}\left(r\right)e^{i\left(k\cdot r\right)}$$

where *r* (*x*, *y*, *z*) is the position vector, and *k* (*k_x_*, *k_y_*, *k_z_*) is the Bloch wave vector. The governing equation of elastic wave combining the boundary conditions, leads to an eigenvalue problem. Thus, the discrete form of eigenvalue equations in the unit can be written as

(5) $$\left(K-\omega^{2}M\right)u=0,$$

where *K* is the stiffness matrices, *M* is mass matrices, *u* is the nodal displacement, and *ω* is the angular frequency. *K* represents the relations between the nodal displacement and force. Only one unit cell needs to be considered based on Bloch theorem. Due to the symmetry, the whole structure star can be considered as a simple cubic lattice as shown in Figure 1b. The structure is assumed to be infinite and periodic in the *x*, *y,* and *z* directions when calculating the band gap. Furthermore, the Bloch—Floquet periodic boundary conditions were applied along the *x*, *y,* and *z* directions [36]:

(6) $$u\left(x+a,y,z\right)=u\left(x,y,z\right)e^{i\left(k_{x}\cdot a\right)}$$

(7) $$u\left(x,y+a,z\right)=u\left(x,y,z\right)e^{i\left(k_{y}\cdot a\right)}$$

(8) $$u\left(x,y,z+a\right)=u\left(x,y,z\right)e^{i\left(k_{z}\cdot a\right)}$$

where *k_x_*, *k_y_*, and *k_z_* are the components of the Bloch wave vector in the *x*, *y,* and *z* directions, respectively, and *a* is the lattice constant. The eigenfrequencies and corresponding vibration modes can be obtained by solving Equation (5) in FEM software. Furthermore, the whole dispersion curves can be calculated by sweeping *k* along the boundaries of the irreducible Brillouin zone as shown in Figure 1b. When calculating transmission loss, the finite elements (four units) are taken in one direction (*x* direction), and the periodic boundary conditions are applied in the other two directions (*y*, *z* directions) to represent infinite units. Assuming the elastic wave was normally incident on the surface of the whole structure in *x* direction, the transmission loss was calculated from transmission coefficient by integral mean of the displacement at interface. Meanwhile, to suppress the wave reflection at the interface, a perfect matching layer (PML) was used in the *x* direction. It is also noted that the maximum element size of the mesh is less than 1/6 of the shortest wavelength in calculated frequency ranges to ensure the accuracy of the calculation.

## 3. Results

### 3.1. Poisson’s Ratio of the 3D Star Structure

**Figure 3:**
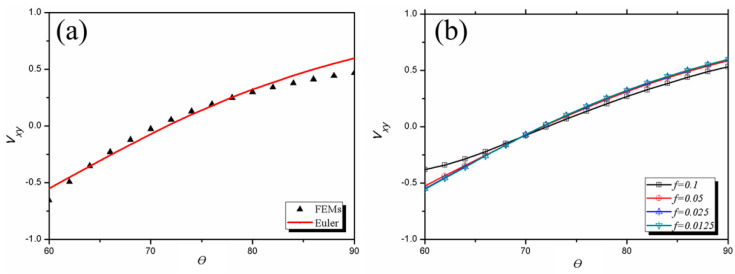
Poisson’s ratio calculated for the star structure versus the angle *θ* (**a**) using the finite element method (FEM) and Euler beam model with *f* = 0.025 and (**b**) at different slenderness ratios *f*.

To simplify the calculations, it was assumed that *L*_1_ is equal to *L*_2_, and *e* = 1. The calculated Poisson’s ratio for the 3D star structure with different angles *θ* and slenderness ratios *f* are presented in Figure 3. The results calculated using the analytical expressions given in Equation (2) agreed well with the FEM results (Figure 3a), especially in the *θ* range of 65° to 80°. This agreement proves the validity of the calculation results in this paper. The auxetic behavior of the 3D star structure is closely related to *θ*, and *θ* = 70° is the critical angle. When *θ* > 70°, the 3D star structure shows no auxetic behavior. When *θ* < 70°, the whole structure shows auxeticity; that is, its Poisson’s ratio is negative. The relationship between Poisson’s ratio and *f* was also investigated, as shown in Figure 3b. When *f* < 0.05, the Poisson’s ratio changed little, which was consistent with the results obtained for a 2D star structure [35,40]. It was found that the auxetic behavior was almost independent of *f* and the critical angle for auxetic behavior was 70° regardless of *f*.

### 3.2. Band Structure of the 3D Star Metamaterials

**Figure 4:**
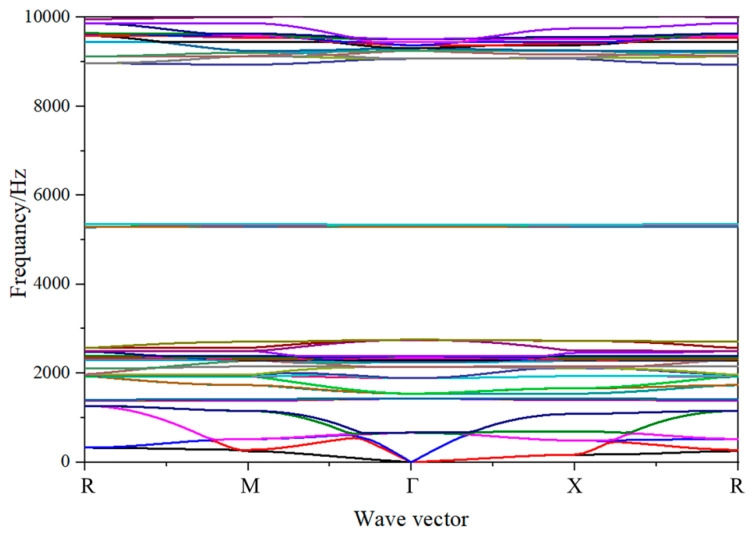
Band gap diagram of the 3D Star structure.

The band structure and vibration mode of the 3D star metamaterials were studied systematically using the FEM. Figure 4 is the calculated result of the band structure while the 3D star unit with a concave angle of 60°. There were many band gaps in the calculated frequency ranges, including the first band gap in the frequency range 1268.6–1382.5 Hz, the second band gap in the frequency range 1430.6–1543.8 Hz, the third band gap in the frequency range 2753.2–5285.7 Hz, and the fourth band gap in the frequency range 5352.7–8937.5 Hz. However, the lattice constant of the star structure was 0.025 m, which is far shorter than the wavelength corresponding to the center frequencies of band gaps. The low-frequency band gap of the 3D star structure originated from local resonance. Different from the 2D case, the 3D star structure has more dispersion curves and band gaps in the studied frequency range because of its abundant modes [35,36]. The dispersion curves of both 2D and 3D structures can form a wide band gap in the low frequency range, although the low-frequency band gap of the 3D star structure is lower than that of the 2D. In addition, the center frequency of the band gap of the 3D structure is about half of that of the 2D case with the same lattice constant. This makes the 3D structure promising for use in low-frequency wide-band elastic wave isolation and filtering.

**Figure 5:**
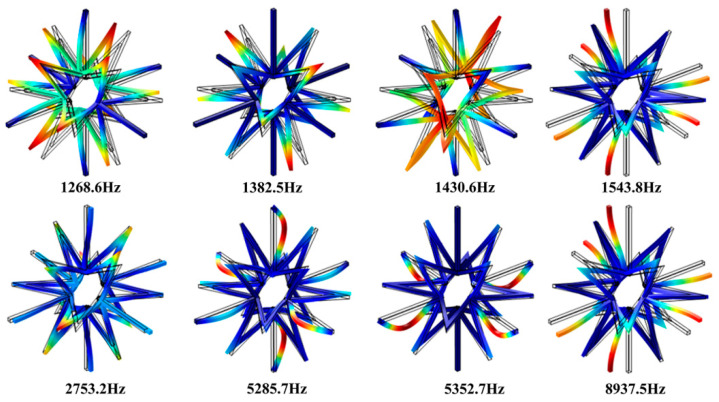
Vibration modes at the band gap cut-off frequencies of the 3D star structure.

To further investigate the physical mechanism of band gap formation in the 3D star structure, the corresponding vibration modes at the cut-off frequencies were identified. Figure 5 depicts the corresponding vibration modes at the band gap cut-off frequencies, where the black lines represent the primitive cell shape, and the colored areas show the shape of the deformed cell. The modal deformation of the 3D star structure is mainly depended on the bending deformation of the rods. It is noted from the vibration modes that the band gap formation of the 3D star structure can be divided into two mechanisms. The first band gap (1268.6–1382.5 Hz) and second band gap (1430.6–1543.8 Hz) are consistent with the deformations mechanism of the first band gap of the 2D case, which is analogous to a traditional three-component local resonant phononic crystal. The six connecting ribs can be approximated as springs, and the star structure in the center can be approximated as a mass point. Because the center mass of the 3D structure is larger than that of the 2D case, the band gap frequency is lower. The third band gap (2753.2–5285.7 Hz) and fourth band gap (5352.7–8937.5 Hz) display a similar formation mechanism to that of the second band gap of the 2D star metamaterials, which is mainly related to the bending of the connecting rib. The corresponding vibration modes of the 3D star structure at the cut-off frequencies are mainly depended on the bending deformations of the connecting ribs and the node ribs. The bending deformations of these ribs lead to the formation of a variety of resonance structures, which is the main reason for the induced band gap.

**Figure 6:**
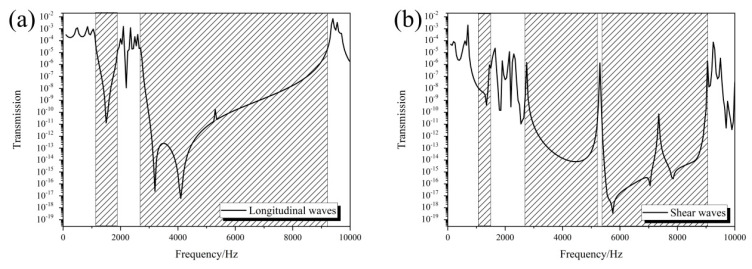
Transmission curve of the 3D star structure for longitudinal waves (**a**), and for shear waves (**b**).

The 3D star structure can form a wide band gap at low frequency, which means that it can be designed as a vibration isolation structure. To further evaluate the band gap and elastic wave isolation characteristics of the 3D star structure, the FEM was used to calculate its transmission characteristics in the ΓΧ direction. In this model, four finite periods were selected in the *x* direction, and periodic boundary conditions were applied in the other two directions (*y* and *z* directions) to simulate the infinite units. In the calculation, the periodic displacement was applied at one end of the structure to make the vibration propagate along the ΓX direction, and the resulting transmission curve of the structure was obtained by dividing the collected displacement at the other end by the displacement at the input end. The transmissions were calculated by using the shear and longitudinal waves. Figure 6a shows the transmission curves of the star structure in the frequency range 10–10,000 Hz along the ΓΧ direction for longitudinal waves. The transmission curve exhibited obvious vibration attenuation in the frequency ranges 1180–1850 Hz and 2800–9200 Hz; that is, band gaps formed in these two frequency ranges, whereas the longitudinal and transverse waves propagated in other frequency ranges. Furthermore, the same results were obtained for the shear waves as shown in Figure 6b. The difference is that there is more pronounced peak around 5300 Hz due to a little gap between the second and third band gap. These findings are consistent with the results of band structures shown in Figure 4. In addition, it is noted that the transmission is low even outside the band gaps due to the impedance mismatch between the matrix and 3D star structure. The calculated transmission characteristics show that the star structure can achieve wide-band isolation of elastic waves in a low frequency range with low mass density, which make it useful for the design of new filters and isolators.

### 3.3. Influence of the Structure Parametres on the Band Gap of the 3D Star Structure

**Figure 7:**
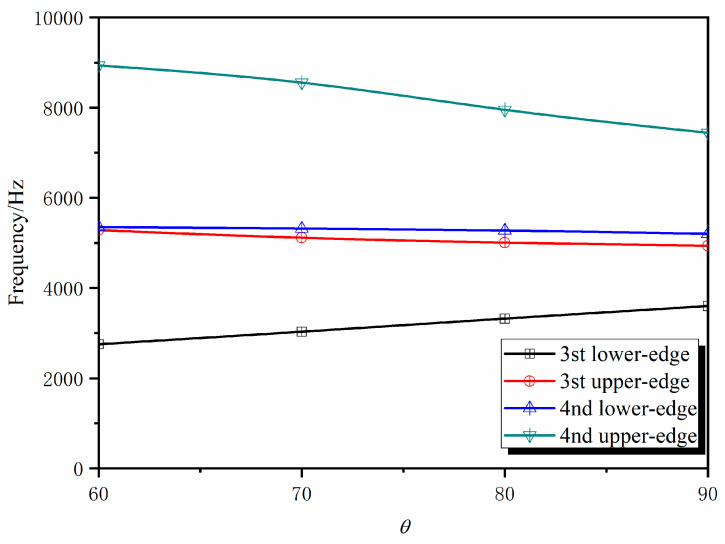
Band gap cut-off frequencies of the 3D star structure at different *θ*.

**Figure 8:**
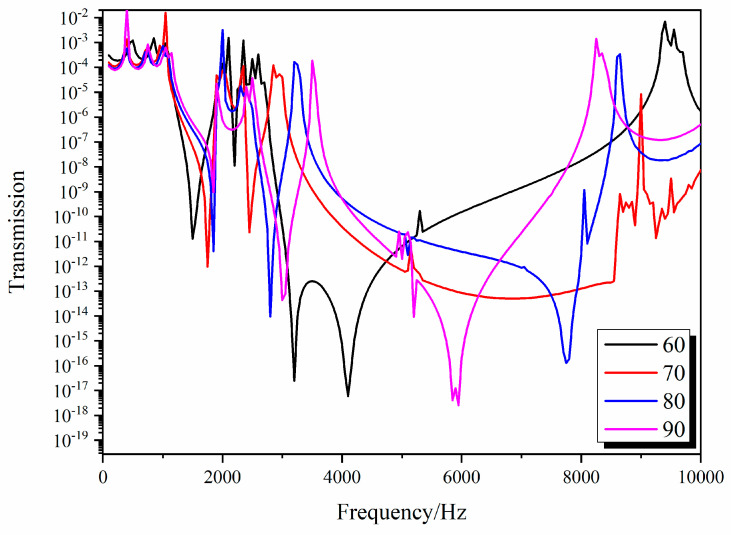
Dependence of the transmission curves of the 3D Star structure on *θ*.

The 3D star metamaterials have a series of structural parameters, including *θ*, *L*_1_, *L*_2_, *t*, and *a*. These structural parameters can change the shape of the structure to adjust its overall mechanical properties and regulate its band structure. The negative Poisson’s ratio calculated for the 3D star structure suggests that *θ* and *f* can effectively control both its Poisson’s ratio and static mechanics. Furthermore, it was assumed that *L*_1_ is equal to *L*_2_. In order to prove that the tunability of the band gap, the influences of *θ* and *f = t/L*_1_ on the wave characteristics of the 3D star structure were investigated. To investigate the influences of concave angle *θ* on the wave characteristics of the 3D star metamaterials, we studied the band structure and transmission curves as *θ* varied from 70° to 90° and analyzed the influence of *θ* on the position and width of the band gaps. In the calculation, only *θ* was changed; *L*_1_ and *t* were kept constant. Figure 7 and Figure 8 show the changes of the third and fourth band gap cut-off frequencies and transmission curves with *θ*, respectively.

Because the third and fourth band gaps are wide and thus have considerable application value in vibration isolation engineering, we only considered the change of the cut-off frequencies of the third and fourth band gaps with *θ*, as shown in Figure 7. The lower cut-off frequency of the third band gap gradually moves to a higher frequency with increasing *θ*, whereas the upper cut-off frequency moves to a lower frequency. The lower cut-off frequency of the fourth band gap remains almost constant as *θ* changes, whereas the upper cut-off frequency moves to lower frequency with increasing *θ*, which also causes the band gap to gradually narrow with increasing *θ*. The dependence of the band gap cut-off frequencies of the 3D star structure on *θ* was similar to that of the 2D structure. The resonance state formed by the bending deformation of the rib plate is conducive to the opening of the band gap. The band structure was influenced by *θ*, which means that the change of *θ* can realize the effective regulation of band structure.

Figure 8 shows the influence of *θ* on the transmission curves of the 3D star structure for longitudinal waves. With increasing *θ*, the transmission loss valleys corresponding to the four band gaps moved to a higher frequency, and the width of the valleys gradually narrowed. This is consistent with the calculated change of band gap with *θ*. By changing *θ*, the vibration of a specific frequency band can be effectively isolated. The best vibration isolation performance was obtained when *θ* was 60°.

**Figure 9:**
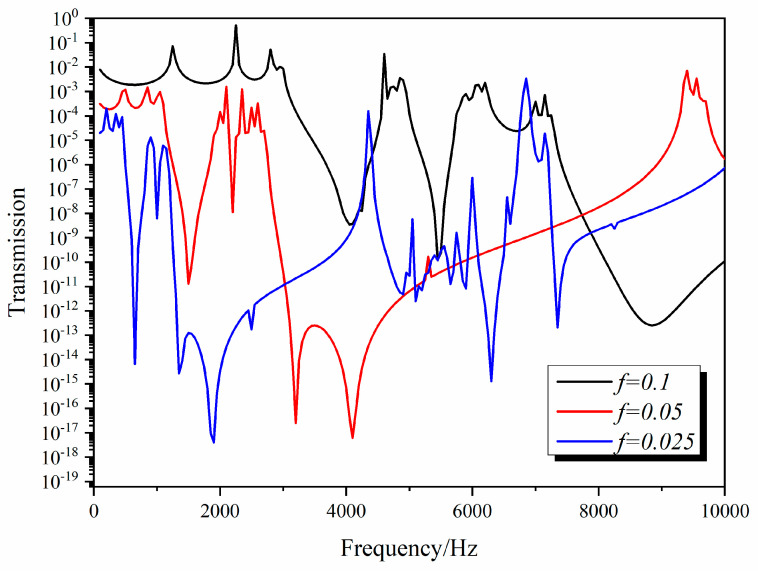
Dependence of the transmission curves of the 3D star structure on *f*.

Figure 9 shows the transmission curve of the 3D star structure changing with *f* longitudinal waves. With increasing *f*, the transmission loss valley corresponding to the band gap moved to a higher frequency. This is because the band gap of the 3D star structure is essentially a local resonance, which is similar to a three-component local resonant phononic crystal. The six connecting ribs can be approximated as springs, and the center of the star structure can be approximated as a mass point. As *f* increases, the stiffness of connecting ribs increases; that is, *K* increases, which makes the center frequency of the band gap move to a higher frequency, so the vibration isolation valley also moves to a higher frequency. These results show that many parameters of the 3D star structure can be effectively isolated by adjusting its structural features and prove that the 3D star structure has a good designability. In additions, it can be predicted that all the frequencies are moving to high frequencies when the scale is reduced to the nanometers, maybe to MHz, which is useful in MEMS.

## 4. Conclusions

The Poisson’s ratio, band structure, and low-frequency vibration isolation performance of a 3D star structure were systematically studied using the FEM. The results showed that the 3D star structure formed a wide band gap at lower frequency and had a negative Poisson’s ratio. Different to conventional acoustic metamaterials, the main physical mechanism behind the low-frequency band gap of the 3D star structure was the resonance mode formed by the bending deformation of each rib plate, which made it easier to achieve effective isolation of low-frequency elastic waves with a low mass density. At the same time, many structural parameters of the 3D star structure can be modulated to effectively adjust the band gap frequency by changing the angle between the concave nodes and the aspect ratio. This study provides a new idea for the design of 3D acoustic metamaterials and the development of new lightweight vibration isolation devices.

## Appendix A

The deformation and stress were able to be obtained using one quarter of the 2D unit cell, as shown in Figure 2b, when an axial force was applied to the structure along the *x* direction. The detailed derivation of the internal forces of the static structure is shown in Figure 2b using the force method. First, the positive direction *x*_1_ was selected for the AB rod and then the positive directions *x*_2_, *x*_3_, and *x*_4_ for the other three rods were selected. When a force *F* = 4*P* in the *x* direction is applied at point *A*, the horizontal force acting at *B* is *P* because of the symmetry of the structure.

The redundant force X_1_ can be obtained by solving equilibrium equations called the force method [35]:

(A1) $$\delta_{11}X_{1}+\Delta_{1P}=0$$

where $\delta_{11}$ is the displacement in the direction of the redundant force under *X*_1_ = 1 and $\Delta_{1P}$ is the displacement under the horizontal force *P*.

According to Mohre’s theorem [35],

(A2) $$\delta_{11}=\int_{0}^{a}\frac{{\bar{M}}_{1}{\bar{M}}_{1}}{EI_{2}}dx=\frac{1}{EI_{2}}(1\times 1\times L_{2}\times 2)=\frac{2L_{2}}{EI_{2}}$$

(A3) $$\Delta_{1P}=\frac{1}{EI_{2}}(\frac{3}{2}PL_{2}{}^{2}\sin\theta -\frac{1}{2}PL_{2}{}^{2}\cos\theta )$$

Substituting Equation (A3) into Equation (A2) allows *X*_1_ to be obtained using Equation (4),

(A4) $$X_{1}=-\frac{\Delta_{1P}}{\delta_{11}}=-\frac{3}{4}PL_{2}\sin\theta +\frac{1}{4}PL_{2}\cos\theta$$

The bending moment distribution in one quarter of the unit cell under the combined action of load and bending moment is:

(A5) $$\mathrm{Beam}\;\mathrm{AB}:\;M_{1}(x_{1})=0\;M_{1}(x_{1})=0\;\left(0\leq x_{1}\leq L_{1}\right)$$

(A6) $$\mathrm{Beam}\;\mathrm{BC}:\;M_{2}(x_{2})=x_{2}P\sin\theta +(-\frac{3}{4}PL_{2}\sin\theta +\frac{1}{4}PL_{2}\cos\theta ),(0\leq x_{2}\leq L_{2})$$

(A7) $$\mathrm{Beam}\;\mathrm{CD}:\;M_{3}(x_{3})=-Px_{3}\cdot \cos\theta +\frac{1}{4}PL_{2}\sin\theta +\frac{1}{4}PL_{2}\cos\theta ,(0\leq x_{3}\leq L_{2})$$

(A8) $$\mathrm{Beam}\;\mathrm{DE}:\;M_{4}(x_{4})=0,\;(0\leq x_{4}\leq L_{1})$$

The analysis of the end point force in one quarter of the unit cell is shown in Figure 2b.

$F_{S}$ indicates the shear force, $F_{N}$ indicates the axial force, the tension along the axis is positive, and the pressure is negative. The axial force and shear force distributions are:

(A9) $$\mathrm{Beam}\;\mathrm{AB}:\;F_{S1}(x_{1})=0,\;F_{N1}(x_{1})=-4P,\;(0\leq x_{1}\leq L_{1}).\infty$$

(A10) $$\mathrm{Beam}\;\mathrm{BC}:\;F_{S2}(x_{2})=P\sin\theta ,\;F_{N2}(x_{2})=P\cos\theta ,\;(0\leq x_{2}\leq L_{2}).$$

(A11) $$\mathrm{Beam}\;\mathrm{CD}:\;F_{S3}(x_{3})=-P\cos\theta ,\;F_{N3}(x_{3})=-P\sin\theta ,\;(0\leq x_{3}\leq L_{2}).$$

(A12) $$\mathrm{Beam}\;\mathrm{DE}:\;F_{S4}(x_{4})=0,\;F_{N4}(x_{4})=0,\;(0\leq x_{4}\leq L_{1})$$

Under force *4P* in the *x* direction, the *x* direction is deformed to

(A13) $$\Delta_{x}={\Delta_{x}}^{b}+{\Delta_{x}}^{s}+{\Delta_{x}}^{c}$$

where ${\Delta_{x}}^{b}$ represents the displacement of the structure along the *x* direction under the action of the bending, ${\Delta_{x}}^{s}$ represents the displacement along the *x* direction under the shear force, ${\Delta_{x}}^{c}$ represents the displacement in the *x* direction under the action of the axial force, and $\tilde{M}$ represents the bending moment in each rod when the horizontal direction unit force acts on the AB rod.

(A14) $$\begin{array}{l} {\Delta_{x}}^{b}=\sum_{i=1,4}\int_{0}^{l}\frac{M_{i}(x_{i}){\tilde{M}}_{i}(x_{i})}{EI_{1}}dx_{i}+\sum_{i=2,3}\int_{0}^{a}\frac{M_{i}(x_{i}){\tilde{M}}_{i}(x_{i})}{EI_{2}}dx_{i}\\ =\frac{P}{EI_{2}}(\frac{5}{96}L_{2}{}^{3}-\frac{1}{16}L_{2}{}^{3}\sin\theta \cos\theta ) \end{array}$$

(A15) $${\Delta_{x}}^{s}=\sum_{i=1,4}\int_{0}^{l}\frac{kF_{si}(x_{i}){\tilde{F}}_{si}(x_{i})}{GA_{1}}dx_{i}+\sum_{i=2,3}\int_{0}^{a}\frac{kF_{si}(x_{i}){\tilde{F}}_{si}(x_{i})}{GA_{2}}dx_{i}=\frac{kPL_{2}}{4GA_{2}}$$

(A16) $${\Delta_{x}}^{c}=\sum_{i=1,4}\int_{0}^{l}\frac{F_{sNi}(x_{i}){\tilde{F}}_{Ni}(x_{i})}{EA_{1}}dx_{i}+\sum_{i=2,3}\int_{0}^{a}\frac{F_{Ni}(x_{i}){\tilde{F}}_{Ni}(x_{i})}{EA_{2}}dx_{i}=\frac{4PL_{1}}{EA_{1}}+\frac{PL_{2}}{EA_{2}}$$

(A17) $$\Delta_{x}=\frac{Pa^{3}}{EI_{2}}(\frac{5}{96}-\frac{1}{16}\sin\theta \cos\theta )+\frac{kPL_{2}}{4GA_{2}}+\frac{4PL_{1}}{EA_{1}}+\frac{PL_{2}}{EA_{2}}$$

The strain of the structure in the *x* direction can be defined by

(A18) $$\varepsilon_{x}=\frac{\Delta_{x}}{\frac{\sin(\theta -\pi /4)}{\sin(\pi /4)}\cdot L_{2}+L_{1}}$$

According to Moore’s theorem, when the unit force is applied in the *y* direction, the bending moment in each rod can be obtained:

(A19) $$\mathrm{Beam}\;\mathrm{AB}:\;{\overset{⌢}{M}}_{1}(x_{1})=0\;\left(0\leq x_{1}\leq L_{1}\right)$$

(A20) $$\mathrm{Beam}\;\mathrm{BC}:\;{\overset{⌢}{M}}_{2}(x_{2})=-\frac{1}{4}x_{2}\cos\theta +\frac{3}{16}L_{2}\cos\theta -\frac{1}{16}L_{2}\sin\theta )(0\leq x_{2}\leq L_{2})$$

(A21) $$\mathrm{Beam}\;\mathrm{CD}:\;{\overset{⌢}{M}}_{3}(x_{3})=\frac{1}{4}x_{3}\cdot \sin\theta -\frac{1}{16}L_{2}\sin\theta -\frac{1}{16}L_{2}\cos\theta ,(0\leq x_{3}\leq L_{2})$$

(A22) $$\mathrm{Beam}\;\mathrm{DE}:\;{\overset{⌢}{M}}_{4}(x_{4})=0,\;(0\leq x_{4}\leq L_{1})$$

The axial force and shear force distributions are:

(A23) $$\mathrm{Beam}\;\mathrm{AB}:\;{\overset{⌢}{F}}_{S1}(x_{1})=0,\;{\overset{⌢}{F}}_{N1}(x_{1})=0,\;(0\leq x_{1}\leq L_{2}).$$

(A24) $$\mathrm{Beam}\;\mathrm{BC}:\;{\overset{⌢}{F}}_{S2}(x_{2})=\frac{1}{4}\cos\theta ,\;{\overset{⌢}{F}}_{N2}(x_{2})=-\frac{1}{4}\sin\theta ,\;(0\leq x_{2}\leq L_{2}).$$

(A25) $$\mathrm{Beam}\;\mathrm{CD}:\;{\overset{⌢}{F}}_{S3}(x_{3})=-\frac{1}{4}\sin\theta ,\;{\overset{⌢}{F}}_{N3}(x_{3})=\frac{1}{4}\cos\theta ,\;(0\leq x_{3}\leq L_{2}).$$

(A26) $$\mathrm{Beam}\;\mathrm{DE}:\;{\overset{⌢}{F}}_{S4}(x_{4})=0,\;{\overset{⌢}{F}}_{N4}(x_{4})=-1,\;(0\leq x_{4}\leq L_{1})$$

Under an applied force in the *y* direction, the *y* direction is deformed to $\Delta_{y}=\Delta_{y}{}^{b}+\Delta_{y}{}^{s}+\Delta_{y}{}^{c}$, where $\Delta_{y}{}^{b}$ represents the displacement of the structure in the *x* direction under the action of the bending moment, $\Delta_{y}{}^{s}$ represents the displacement in the *x* direction under the shear force, and $\Delta_{y}{}^{c}$ represents the displacement in the *x* direction under the action of the axial force.

(A27) $$\begin{array}{l} \Delta_{y}=\Delta_{y}{}^{b}+\Delta_{y}{}^{s}+\Delta_{y}{}^{c}=\\ \sum_{i=1,4}\int_{0}^{l}\frac{M_{i}(x_{i}){\overset{⌢}{M}}_{i}(x_{i})}{EI_{1}}+\frac{k{\overset{⌢}{F}}_{si}(x_{i}){\overset{⌢}{F}}_{si}(x_{i})}{GA_{1}}+\frac{{\overset{⌢}{F}}_{Ni}(x_{i}){\overset{⌢}{F}}_{Ni}(x_{i})}{EA_{1}}dx_{i}+\\ \sum_{i=2,3}\int_{0}^{a}\frac{M_{i}(x_{i}){\overset{⌢}{M}}_{i}(x_{i})}{EI_{2}}+\frac{k{\overset{⌢}{F}}_{si}(x_{i}){\overset{⌢}{F}}_{si}(x_{i})}{GA_{2}}+\frac{{\overset{⌢}{F}}_{Ni}(x_{i}){\overset{⌢}{F}}_{Ni}(x_{i})}{EA_{2}}dx_{i}\\ =\frac{PL_{2}{}^{3}}{EI_{2}}(\frac{1}{32}-\frac{5}{48}\sin\theta \cos\theta )+\frac{PkL_{2}}{2GA_{2}}\sin\theta \cos\theta -\frac{2PL_{2}}{EA_{2}}\sin\theta \cos\theta \end{array}$$

Under the action of the horizontal force 4*P* in the *x* direction, the strain in the *y* direction of the structure is:

(A28) $$\varepsilon_{y}=\frac{\Delta_{y}}{\frac{\sin(\theta -\pi /4)}{\sin(\pi /4)}\cdot L_{2}+L_{1}}$$

The equivalent Poisson’s ratio *v*_12_ is:

(A29) $$\nu_{12}=-\frac{\varepsilon_{y}}{\varepsilon_{x}}=-\frac{\frac{PL_{1}{}^{3}}{EI_{1}}(\frac{1}{32}-\frac{5}{48}\sin\theta \cos\theta )+\frac{PkL_{1}}{2GA_{1}}\sin\theta \cos\theta -\frac{2PL_{2}}{EA_{2}}\sin\theta \cos\theta }{\frac{PL_{2}{}^{3}}{EI_{2}}(\frac{5}{96}-\frac{1}{16}\sin\theta \cos\theta )+\frac{kPL_{2}}{4GA_{2}}+\frac{4PL_{1}}{EA_{1}}+\frac{PL_{2}}{EA_{2}}}$$

When there is zero shear in the Euler beam model, *k* = 0, $e=\frac{L_{2}}{L_{1}},f=\frac{t}{L_{1}}$, which means that *v*_12_ can be described by:

(A30) $$\nu_{12}=-\frac{\varepsilon_{y}}{\varepsilon_{x}}=\frac{(\frac{3}{8}-\frac{5}{4}\sin\theta \cos\theta )e^{3}-2ef^{2}\sin\theta \cos\theta }{(\frac{5}{8}-\frac{3}{4}\sin\theta \cos\theta )e^{3}+4f^{2}+ef^{2}}$$
